# Progression of a Rare Disease, Takayasu Arteritis, With Hematologic and Gastrointestinal Manifestations: A Four-Year Follow-Up Study

**DOI:** 10.7759/cureus.45950

**Published:** 2023-09-25

**Authors:** Daniel Hernan Sacoto, Valentina Turbay-Caballero, Tiago Reyes-Castro, Bryan S Quintanilla, Delatre Lolo

**Affiliations:** 1 Internal Medicine, Metropolitan Medical Center, New York, USA; 2 Internal Medicine, Advocate Christ Medical Center, Chicago, USA; 3 Internal Medicine, New York Medical College, Metropolitan Hospital Center, New York, USA

**Keywords:** large vessel vasculitis, eosinophilic esophagitis, monoclonal gammopathy of undetermined significance (mgus), dieulafoy’s ulcer, takayasu arteritits

## Abstract

Takayasu arteritis (TA) is a heterogeneous disease whose presentation and progression have not yet been well described. An elderly female was diagnosed with TA after presenting with bilateral arm claudication, elevated ESR, and bilateral subclavian arterial stenosis. In the first two years after diagnosis, she was diagnosed with monoclonal gammopathy of undetermined significance and alpha thalassemia minor. For the next two years, she presented with a non-ST elevation myocardial infarction, three oozing Dieulafoy lesions, and eosinophilic esophagitis. As we observed, TA can have an unusual and unpredictable progression. Therefore, a multidisciplinary approach and clinical surveillance are paramount.

## Introduction

Takayasu arteritis (TA) is a rare inflammatory systemic vasculitis affecting medium and large arteries, with a particular predilection for the aorta and its major branches, including the subclavian and extracranial arteries (60-90%) [1]. The disease primarily targets women of childbearing age, although the age at presentation can vary widely, ranging from 4 to 63 years. Fewer than 15% of cases occur in individuals older than 40 years. The highest incidence is observed in Asia, while in the United States, TA is considered a rare disease with an estimated incidence of two to three cases per million people per year [2]. While it is established that the pathologic process of TA involves all arterial layers, the disease is still considered heterogeneous, as the pattern of presentation and progression remains incompletely described [3]. Clinical manifestations of TA can range from vascular symptoms in the pulseless stage to nonspecific constitutional signs or symptoms arising from organs whose arteries are compromised by the disease [4]. We present a unique case of TA with multiple organ manifestations over a four-year follow-up period.

## Case presentation

**Figure 1:**
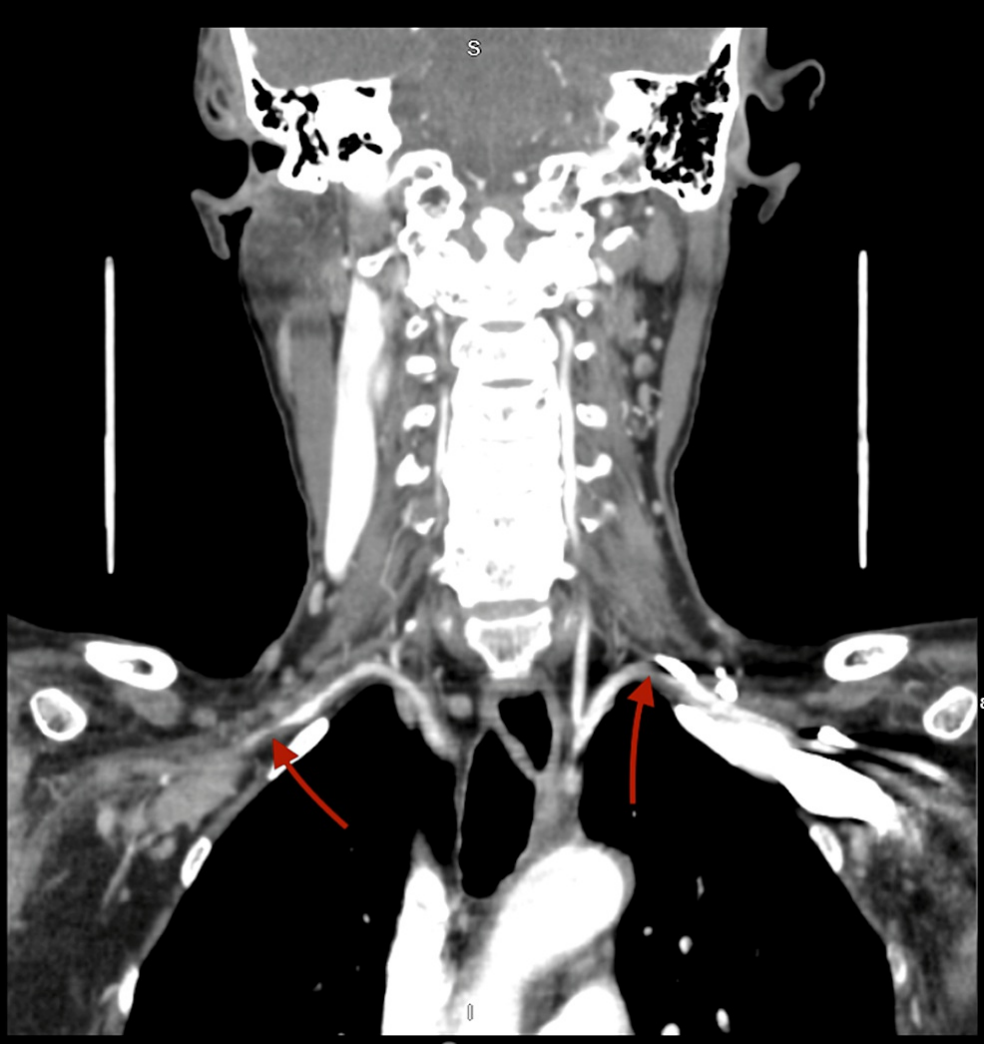
Bilateral subclavian arterial thickening and stenosis.

**Figure 2:**
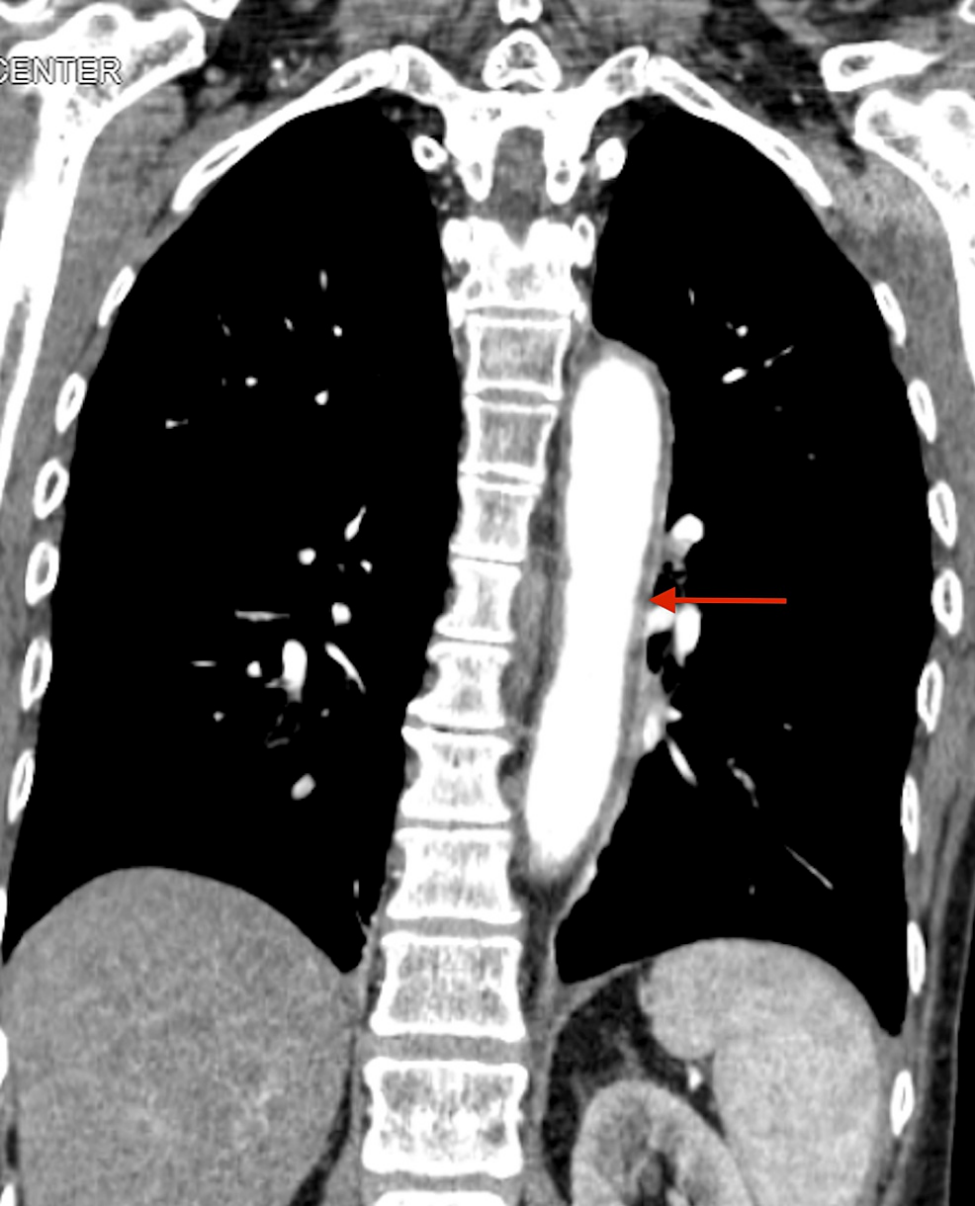
Thoracoabdominal arterial thickening.

A 74-year-old female was diagnosed with TA four years ago after presenting with bilateral arm claudication, reduced pulse on bilateral radial arteries, and systolic blood pressure difference between upper (85/62 mmHg) and lower extremities (132/76 mmHg). At that time, the erythrocyte sedimentation rate was elevated (112 mg/dL). Computed tomography of the neck-chest-abdomen-pelvis with contrast showed bilateral subclavian artery stenosis (Figure 1) and diffuse thoracoabdominal arterial wall thickening (Figure 2).

 

**Figure 3:**
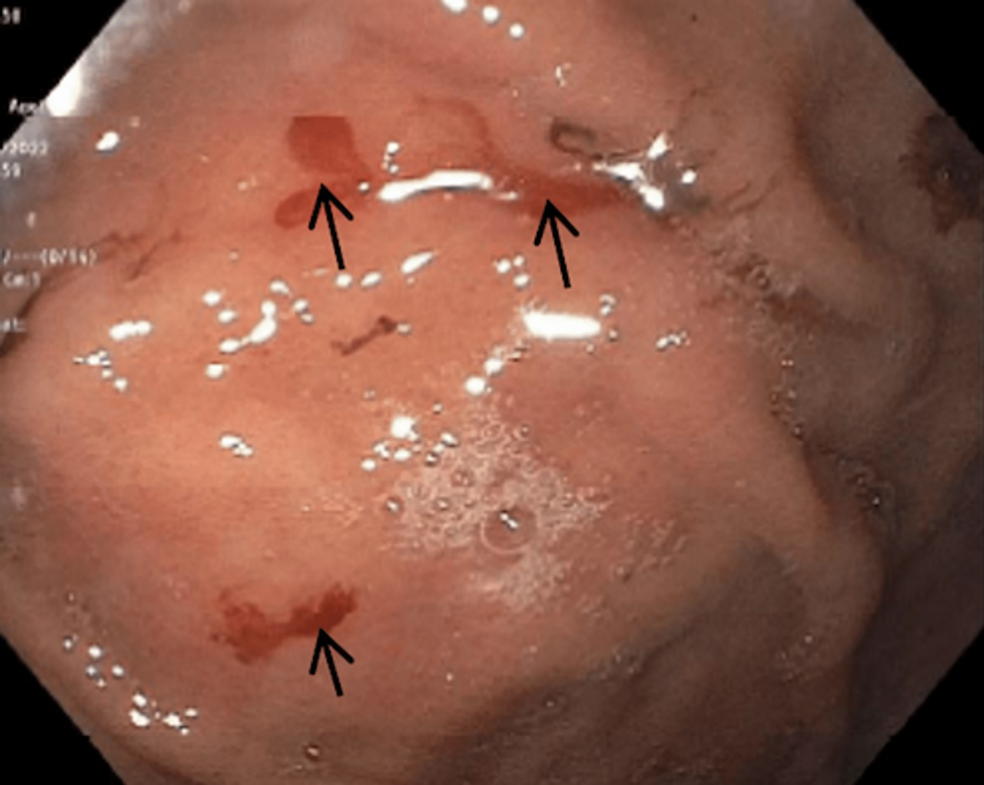
Lesser curvature of the stomach showing three oozing Dieulafoy lesions.

**Figure 4:**
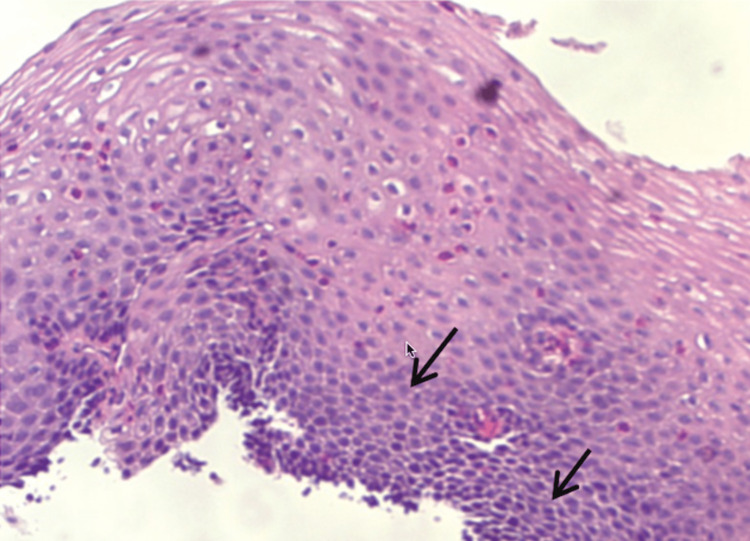
Eosinophilic infiltration of the esophageal mucosa.

Subsequent aortic angiogram confirmed right (70-80%) and left (90-95%) subclavian stenosis. Even though her age at the time of diagnosis was above range, she met a total score of 12 points based on the 2022 American College of Rheumatology TA criteria [5]. The patient was started on prednisone (40 mg/daily) in addition to methotrexate 25 mg weekly dosages with subsequent improvement of claudication. An elevated total protein (8.5 g/dL) was noted one year later. Extension workup, including serum protein electrophoresis, showed a gamma-migrating paraprotein (1.8 g/dL) with a predominant IgG subtype. Hypercalcemia, renal insufficiency, or lytic lesions were absent; therefore, a non-IgM Monoclonal Gammopathy of Undetermined Significance (MGUS) was established. Two years later, follow-up laboratories showed microcytic anemia (Hb: 10.2 g/dL; MCV: 71.6 g/dL) with normal levels of iron (37 g/dL), and ferritin (54 ng/dL). Further investigation, including hemoglobin electrophoresis, demonstrated a homozygote loss of alpha chains, compatible with alpha thalassemia minor. DNA analysis confirmed an alpha 3.7 deletion genotype. Three years later, she came with complaints of shortness of breath and retrosternal chest pain. ECG showed new deep anterolateral T wave inversion and elevated Troponin T (0.49 ng/mL). A transthoracic echocardiogram demonstrated a newly reduced ejection fraction (30-35%). She was referred for cardiac catheterization and coronary angiography for non-ST elevation myocardial infarction evaluation. However, there was non-obstructive coronary artery disease with no significant atherosclerosis, characteristic of TA. The case was medically managed with a focus on secondary prevention. Four years later, she started with progressive dysphagia, first to solids followed by liquids. A subsequent esophagogastroduodenoscopy found three oozing Dieulafoy lesions on the lesser curvature of the stomach, which were successfully treated with hemostatic clipping (Figure 3). Biopsies retrieved from the esophagus revealed >15 intraepithelial eosinophils in a high-power field (Figure 4), leading to an eosinophilic esophagitis diagnosis. She was started on a proton pump inhibitor and dietary modifications with overall improvement.

 

## Discussion

**Figure 5:**
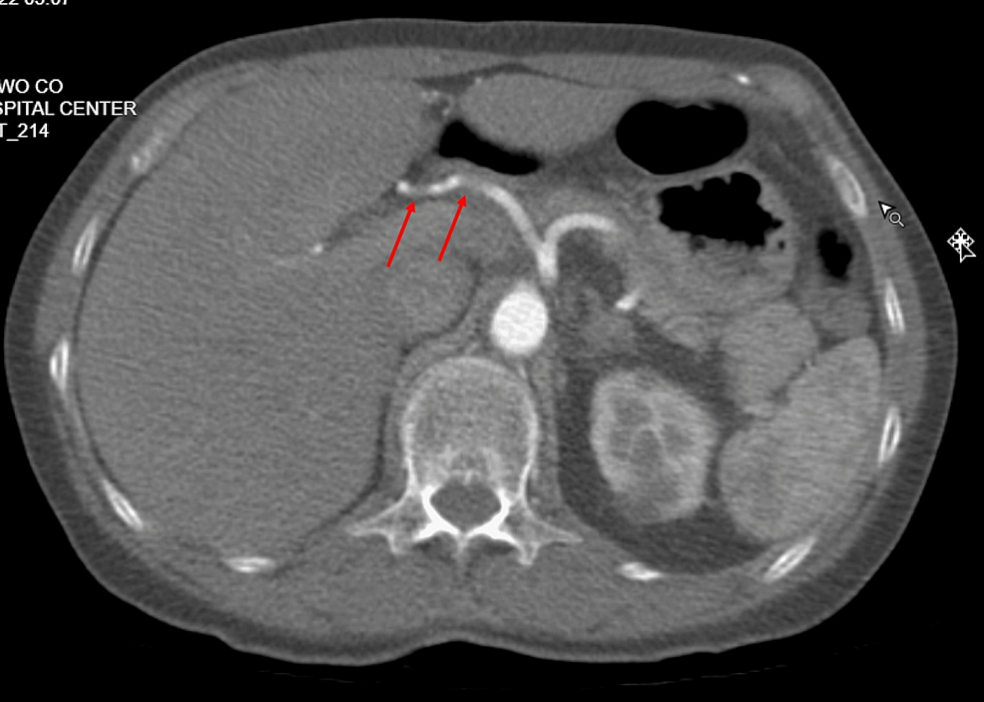
Narrowing of celiac artery branches.

Clinical presentation and progression of TA are uncertain [4]. However, it is important to acknowledge all possibilities since complications from vascular damage in different organs can result in substantial morbidity. Regarding associated hematologic conditions, no previous cases have reported an association between MGUS and TA, though it has been proposed that sustained immune stimulation of B-lymphocytes present in autoimmune disorders may lead to clonal proliferation and ultimately to MGUS [6]. Since it carries a 1% annual risk of progression to multiple myeloma, increased awareness will likely benefit those with TA. On the other hand, anemia is a common comorbidity of TA since is associated with the active disease. However, thalassemia in the setting of TA is rare, as only two cases have been reported. The first is associated with beta-thalassemia, and the second with hemoglobin Evanston (alpha 14 deletion) [7,8]. This is the first case of concomitant TA and alpha 3.7 deletion thalassemia. Studies have demonstrated increased levels of adhesion molecules present in the circulating blood of alpha- and beta-thalassemic patients produced by the damaged endothelium, suggesting this mechanism is a potential etiology [9]. Dieulafoy lesions are rare gastrointestinal vascular anomalies [10]. The putative mechanism of TA involves the inflammation of the submucosal plexus of larger vessels in the lesser curvature of the stomach [11]. In our case, this mechanism may be inferred since there was an involvement of the celiac artery, which supplies blood to the lesser curvature of the stomach (Figure 5). Based on our literature review, only one case has previously shown an association between TA and Dieulafoy lesion [12]. In that case, the lesion in a patient manifesting pulmonary Takayasu's arteritis was localized on the esophagus. The importance relies on its recognition since it is known to cause massive bleeding with a previously reported mortality of up to 80% [13]. Our literature review identified just one case of TA and eosinophilic gastrointestinal disease, and was proposed that overactivation of Th17 immunity may represent a shared mechanism in the pathogenesis of both conditions [14]. To our knowledge, this is the first case reported of TA and eosinophilic esophagitis. The importance lies in the accumulation of more cases to clarify the immunological alterations between TA and eosinophilic esophagitis. Finally, we might not forget the risk of coronary involvement, which is the main cause of death in patients with TA. Is of such relevance since can have a rapid progression even if previously seen in “normal” coronary arteries [15]. Therefore, a high degree of suspicion and early medical therapy involving empirical immunosuppressive therapy in addition to secondary prevention is essential. 

## Conclusions

Patients suffering from TA have an unpredictable and unusual progression, where heterogeneity is manifested as multiple organ manifestations over the years. In order to avoid future complications, we consider that a multidisciplinary approach, including regular clinical surveillance, is paramount for a better outcome in this patient population. Clinicians should be aware that hematologic conditions can coexist in patients with TA; then, if necessary, appropriate screening or monitoring can be considered. Recognition of uncommon gastrointestinal manifestations, including Dieulafoy lesions and eosinophilic esophagitis in patients with TA is important due to their potential for significant morbidity and mortality. 
